# Supplementary material for: Common neural choice signals can emerge artefactually amid multiple distinct value signals
Source: Nat Hum Behav. 2024 Sep 6;8(11):2194–208. doi: 10.1038/s41562-024-01971-z (PMC11576515; doi:10.1038/s41562-024-01971-z)
Supplement: Supplementary file 2 — Reporting Summary [file 41562_2024_1971_MOESM2_ESM.pdf]

nature portfolio

Corresponding author(s): Romy Froemer

Last updated by author(s): 07/05/2024

Reporting Summary

Nature Portfolio wishes to improve the reproducibility of the work that we publish. This form provides structure for consistency and transparency in reporting. For further information on Nature Portfolio policies, see our Editorial Policies and the Editorial Policy Checklist.

Statistics

For all statistical analyses, confirm that the following items are present in the figure legend, table legend, main text, or Methods section.

n/a	Confirmed	
☐	☒	The exact sample size (*n*) for each experimental group/condition, given as a discrete number and unit of measurement
☐	☒	A statement on whether measurements were taken from distinct samples or whether the same sample was measured repeatedly
☐	☒	The statistical test(s) used AND whether they are one- or two-sided<br>*Only common tests should be described solely by name; describe more complex techniques in the Methods section.*
☐	☒	A description of all covariates tested
☐	☒	A description of any assumptions or corrections, such as tests of normality and adjustment for multiple comparisons
☐	☒	A full description of the statistical parameters including central tendency (e.g. means) or other basic estimates (e.g. regression coefficient) AND variation (e.g. standard deviation) or associated estimates of uncertainty (e.g. confidence intervals)
☐	☒	For null hypothesis testing, the test statistic (e.g. *F*, *t*, *r*) with confidence intervals, effect sizes, degrees of freedom and *P* value noted<br>*Give P values as exact values whenever suitable.*
☒	☐	For Bayesian analysis, information on the choice of priors and Markov chain Monte Carlo settings
☒	☐	For hierarchical and complex designs, identification of the appropriate level for tests and full reporting of outcomes
☒	☐	Estimates of effect sizes (e.g. Cohen's *d*, Pearson's *r*), indicating how they were calculated

Our web collection on statistics for biologists contains articles on many of the points above.

Software and code

Policy information about availability of computer code

Data collection	Data were collected using Psychopphysics toolbox (Version 3) for Matlab and Matlab (2016b).
Data analysis	Data were analyzed using Matlab (Version 2022b), EEGLAB (Version 13_6_5b), R(4.2.2 (2022-10-31)), Rstudio (Version 2022.12.0+353 (2022.12.0+353)), unfold for matlab (Version 3) and Unfold for Julia (Version 0.4.1), lme4 package (Version 1.1-31), sjPlot package (Version 2.8.12), analysis code is available under https://github.com/froemero/Common_Neural_Choice_Signals_emerge_artifactually

For manuscripts utilizing custom algorithms or software that are central to the research but not yet described in published literature, software must be made available to editors and reviewers. We strongly encourage code deposition in a community repository (e.g. GitHub). See the Nature Portfolio guidelines for submitting code & software for further information.

Data

Policy information about availability of data

All manuscripts must include a data availability statement. This statement should provide the following information, where applicable:

- Accession codes, unique identifiers, or web links for publicly available datasets
- A description of any restrictions on data availability
- For clinical datasets or third party data, please ensure that the statement adheres to our policy

Data for Studies 1, 2 (only data used here), 3 and 4 are available through on different platforms with links provided through github under: https://github.com/froemero/Common_Neural_Choice_Signals_emerge_artifactually.

Human research participants

Policy information about studies involving human research participants and Sex and Gender in Research.

Reporting on sex and gender	We collected gender information and report it as follows: Study 1:The final sample consisted of 39 participants, (27 female). Study 2: The sample in study 2 comprised 39 participants recruited from Brown University and the general community. Participants (26 female, mean age 23.92, SD = 5.14), gave informed consent and received $10 or $15 per hour for their participation.<br>We do not report gender for the reanalyzed datasets, but this information can be found in the original publications.<br>Gender is recorded in the original source data, but not considered as a relevant factor for the present study.
Population characteristics	Study 1: The final sample consisted of 39 participants, (27 female) with a mean age of 20.84 years (SD = 3.90). Study 2: The sample in study 2 comprised 39 participants recruited from Brown University and the general community. Participants (26 female, mean age 23.92, SD = 5.14), gave informed consent and received $10 or $15 per hour for their participation.<br>Health status information was not assessed.
Recruitment	Participants for study 1 and 2 were recruited from Brown University and the general community. Participants signed up in response to advertisements and through SONA (an experiment sign-up software). It is likely that participants signing up for EEG experiments are generally more active and interested in science. Since we are recruiting from a Psychology participant pool, we also have more female than male participants. Since we are not investigating individual differences and only study within subject effects, it is unlikely that any self-selection characteristics have impacted the results.
Ethics oversight	Study 1 & 2 were approved by Brown University's IRB.

Note that full information on the approval of the study protocol must also be provided in the manuscript.

Field-specific reporting

Please select the one below that is the best fit for your research. If you are not sure, read the appropriate sections before making your selection.

☐ Life sciences ☒ Behavioural & social sciences ☐ Ecological, evolutionary & environmental sciences

For a reference copy of the document with all sections, see nature.com/documents/nr-reporting-summary-flat.pdf

Behavioural & social sciences study design

All studies must disclose on these points even when the disclosure is negative.

Study description	The studies are quantitative experimental within-subject designs.
Research sample	See population characteristics above.<br>Participants were recruited from Brown and the general community. The sample is not representative of the general community since it oversamples Brown undergrads and females in particular.<br>Study 1: The final sample consisted of 39 participants, (27 female) with a mean age of 20.84 years (SD = 3.90). Study 2: The sample in study 2 comprised 39 participants recruited from Brown University and the general community. Participants (26 female, mean age 23.92, SD = 5.14), gave informed consent and received $10 or $15 per hour for their participation.<br>Health status information was not assessed.
Sampling strategy	Participants were sampled pseudo randomly (they signed up). The sample sizes were based on previous studies in this line of work, i.e., 30 Shenhav & Karmarkar (2019). We oversampled relative to this study to assure sufficient observations for linear mixed effects models.
Data collection	Data were collected using a computerized experiment and EEG recording equipment. No people were present besides the participant and the experimenter(s). The participant performed the tasks on their own following intermittent instructions. The experimenters were not blind to the study hypotheses, but since choice sets were determines individually, they were blind to individual choice conditions.
Timing	Study 1 data collection: June 2017 through October 2017<br>Study 2 data collection: April 2022 through June 2022
Data exclusions	For the main study 48 (Study 1) participants were recruited from Brown University and the general community. Of these 9 had to be excluded due to technical problems during data acquisition.
Non-participation	No participants dropped out.
Randomization	N/A this is not a between subject design

Reporting for specific materials, systems and methods

We require information from authors about some types of materials, experimental systems and methods used in many studies. Here, indicate whether each material, system or method listed is relevant to your study. If you are not sure if a list item applies to your research, read the appropriate section before selecting a response.

Materials & experimental systems

n/a	Involved in the study	
☒	☐	Antibodies
☒	☐	Eukaryotic cell lines
☒	☐	Palaeontology and archaeology
☒	☐	Animals and other organisms
☒	☐	Clinical data
☒	☐	Dual use research of concern

Methods

n/a	Involved in the study	
☒	☐	ChIP-seq
☒	☐	Flow cytometry
☒	☐	MRI-based neuroimaging
